# Complex Management of an Infected Pancreatic Collection With Walled-Off Necrosis in Acute Pancreatitis: A Case Report

**DOI:** 10.7759/cureus.100596

**Published:** 2026-01-02

**Authors:** João Fustiga, Teresa Miranda, Hugo Moreira, David Nora

**Affiliations:** 1 Department of Intensive Care, Unidade Local de Saúde de Lisboa Ocidental, Lisbon, PRT

**Keywords:** acute necrotising pancreatitis, endoscopic transluminal drainage, polymicrobial infection, sepsis and septic shock, source control

## Abstract

A 38-year-old man, post a 17-day hospitalization for severe necrotizing pancreatitis (NP) with complications of a pancreatic collection and splenic vein thrombosis, presented to the emergency department with severe epigastric pain. Despite initial stability, abdominopelvic angio-CT revealed an enlarged pancreatic collection with active bleeding. Hemorrhagic shock ensued, leading to ICU admission and subsequent multiorgan failure, elevated intra-abdominal pressure, and suspected pancreatic collection infection. Endoscopic transluminal drainage (ETD) was performed, followed by empiric antibiotics, later adjusted to cefuroxime based on collection fluid cultures. The patient developed septic shock with Pseudomonas aeruginosa isolation in blood cultures, prompting targeted ceftazidime therapy. Repeat ETD with necrosectomy addressed the ongoing infection, with polymicrobial cultures necessitating an extended course of targeted antibiotics and antifungals. Clinical improvement ensued, culminating in discharge home. This case underscores the challenges in managing severe NP and highlights the efficacy of a multidisciplinary approach for improved patient outcomes.

## Introduction

Acute pancreatitis (AP) is one of the most frequent gastrointestinal diseases requiring acute hospitalization in the Western world, and its incidence is increasing worldwide [1-4]. AP is an initially sterile inflammation of the pancreas that evokes a systemic inflammatory response syndrome (SIRS) with large heterogeneity in terms of severity [1-5]. Most patients (80%) experience mild symptoms that require only supportive therapy, including fluids, analgesia, and resumption of diet [1-5]. Nevertheless, a small fraction of patients develop severe pancreatitis, defined as pancreatitis with persistent organ failure and/or local complications, requiring admission to intensive care units (ICUs) within the first days due to an overwhelming SIRS response [1-4].

Based on diagnostic imaging, AP can be classified as either interstitial edematous pancreatitis or necrotizing pancreatitis (NP) [1-6]. In addition, the resulting local complications are categorized into four types of collections: acute peripancreatic fluid collections, pseudocysts, acute necrotic collections, and walled-off necrosis [6]. NP is a severe form of AP, with a risk of bleeding and infection [1-6]. The presence of extraluminal gas within a peripancreatic collection on abdominopelvic computed tomography (CT) is highly suggestive of infected pancreatic necrosis. Management options include surgical approaches, such as percutaneous drainage or video-assisted retroperitoneal debridement, as well as endoscopic interventions, including endoscopic transluminal drainage (ETD) or endoscopic transluminal necrosectomy (ETN) [5-10].

The authors present a case of severe septic shock associated with infection and hemorrhage of a necrotic pancreatic collection, requiring repeated ETDs, with complete radiologic resolution at six-month follow-up after hospital discharge.

## Case presentation

A man in his 30s presented to the emergency department with severe epigastric pain and vomiting. His medical history included arterial hypertension managed with perindopril, anxiety disorder treated with escitalopram, and spinal osteoarthritis treated with paracetamol, metamizole, and tramadol as needed. Despite being hemodynamically stable, he was anxious and exhibited tenderness upon palpation of the epigastric region. Laboratory tests revealed an elevated white blood cell count with a predominance of polymorphonuclear cells and high lipase levels. Abdominal ultrasound showed gallbladder sludge with microlithiasis, whereas evaluation of the pancreas was difficult due to gas overlapping. Abdominopelvic-CT showed a globular pancreas, particularly in the tail region, with a maximum thickness of 50 mm, increased density of the retroperitoneal peripancreatic adipose planes extending to the root of the mesentery, and associated mesenteric adenitis.

The diagnosis of AP was established, and the patient rapidly developed sinus tachycardia, polypnea, and hypoxemia associated with uncontrolled pain. He was admitted to the ICU requiring high-flow nasal cannula oxygen therapy and close monitoring for pain management. Ranson’s criteria on admission were 0, increasing to 3 points after 48 hours.

**Figure 1:**
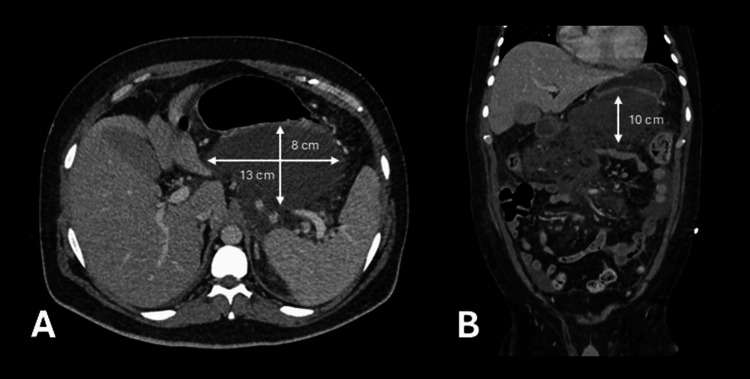
Abdominopelvic CT scan one week after symptom onset. Unfavorable progression with 50% pancreatic necrosis (CT Severity Index: 9) and a large pancreatic collection measuring 13 × 8 × 10 cm, displayed in axial (A) and coronal (B) planes.

In the ICU, the patient maintained hemodynamic stability but experienced extreme pain, requiring multimodal analgesia through epidural and intravenous infusions. Respiratory distress improved, allowing de-escalation of oxygen therapy. A follow-up abdominal CT after one week revealed pancreatic necrosis involving over 50% of the pancreas, a pancreatic collection measuring 13 x 8 x 10 cm (CT Severity Index 9), and partial splenic vein thrombosis, leading to the initiation of a therapeutic dose of low-molecular-weight heparin (enoxaparin) (Figure 1). He was discharged from the ICU after 10 days and remained clinically stable in the surgery department, eventually being discharged home after 17 days on daily enoxaparin.

**Figure 2:**
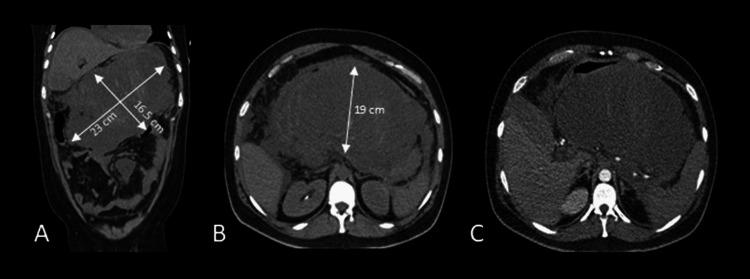
CT progression of necrotizing pancreatitis. Abdominopelvic CT scan showing disease progression, with over 80% pancreatic necrosis (CT Severity Index: 10) and a large acute necrotic collection measuring 23 × 16.5 × 19 cm, visible in coronal (A) and axial (B) planes. Angio-CT confirms active bleeding within the necrotic collection, as seen in the axial plane (C).

Three days after discharge and three weeks after symptom onset, the patient returned to the emergency department with new-onset severe epigastric pain. Initially, he was hemodynamically stable and tender upon abdominal palpation. Laboratory results showed an elevated white blood cell count, with a predominance of polymorphonuclear cells, and high C-reactive protein levels. Amilase and lipase levels were 1243 U/L and 2395 U/L, respectively. Abdominopelvic angio-CT revealed an enlarged pancreatic collection (23 x 16 x 19 cm) with active bleeding (Figure 2). He developed hemorrhagic shock, requiring erythrocyte transfusions, and was readmitted to the ICU with multiorgan failure necessitating orotracheal intubation, mechanical ventilation, vasopressor support (maximum noradrenaline dose of 0.7 mcg/kg/minute), and AKIN 3 acute kidney injury requiring continuous venovenous hemodialysis.

**Figure 3:**
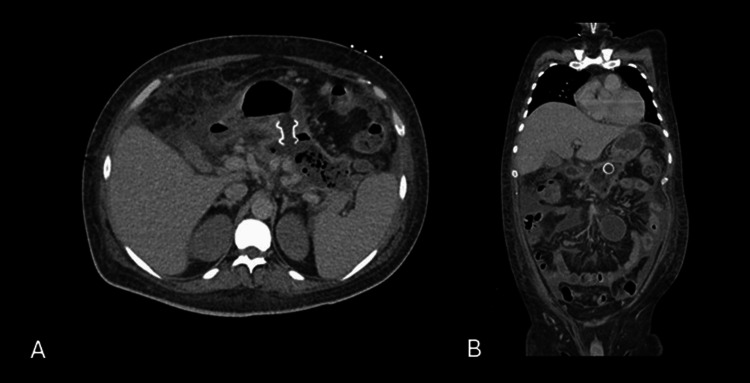
CT progression of necrotizing pancreatitis post-ETD. Post-first endoscopic transluminal drainage (ETD), axial (A) and coronal (B) CT images show a lumen-apposing metal stent (LAMS) placed through the posterior gastric wall into the necrotic collection, with a marked reduction in collection size.

Initial resuscitation, guided by rotational thromboelastometry and supported by blood product transfusion, allowed discontinuation of vasopressors by the third day after ICU admission. Although there were no signs of re-bleeding, the patient’s condition deteriorated within a week with increased intra-abdominal pressure and suspected pancreatic collection infection. Blood cultures collected were negative. ETD with a lumen-apposing covered self-expanding metal stent was performed four weeks after symptom onset to achieve source control (Figure 3). Empirical ceftriaxone and metronidazole were initiated on the same day as the source control procedure; three days later, therapy was de-escalated to cefuroxime after fluid cultures revealed multidrug-sensitive Klebsiella pneumoniae. 

ETD with necrosectomy was repeated four times for source control during the fourth and fifth weeks after initial symptoms, with subsequent fluid cultures revealing polymicrobial infection, including ESBL-producing Klebsiella pneumoniae, Klebsiella aerogenes, Enterococcus faecalis, and Candida glabrata (Candida Score 4). The patient received targeted therapy with meropenem and anidulafungin, showing clinical improvement after completing a 24-day course of antimicrobial therapy. Successive blood cultures were negative, without any suggestion of an extra-abdominal infection focus.

The patient was discharged from the ICU after 51 days and home after 64 days of hospitalization. Six months post-discharge, he did not exhibit any endocrine or exocrine pancreatic deficiencies, and imaging showed the resolution of both the pancreatic collection and vein thrombosis.

## Discussion

Managing AP complicated by a polymicrobial infected pancreatic collection and walled-off necrosis is both challenging and evolving. Infection of the peri-pancreatic collection must be suspected when clinical deterioration occurs, associated with fever and an increase in intra-abdominal pressure. Persistent high C-reactive protein after the first 72 hours appears to be high enough for clinical prediction of infection in pancreatic collections [11]. Extraluminal gas within a (peri)pancreatic collection on CT has a high specificity of 97% for infected necrosis, but a sensitivity of only 56%, indicating that approximately half of patients with infected necrosis will demonstrate gas formation [6]. After diagnosis is made, antibiotic regimens must be initiated covering common gut bacteria and the local resistance patterns, while ETD often serves as an important method for obtaining a culture sample and achieving source control.

Current guidelines advise against the routine use of prophylactic antibiotics for the treatment of AP, since there is no benefit [2-4]. Prophylactic antibiotics may reduce the rate of infection in pancreatic collections, but are not associated with any significant effect on overall mortality [12]. The organisms most commonly cultured are gut bacteria, including members of the Enterobacteriaceae such as Escherichia coli (20%) and Enterobacter species (10%), gram-positive organisms such as Enterococcus faecalis and Enterococcus faecium (22.5% and 20%), and anaerobes (12.5%) [13]. Prolonged treatment with multiple broad-spectrum antibiotics is known to cause a profound alteration in the endogenous flora, promoting the growth of Candida species [6]. Fungal infections are more difficult to detect than bacterial infections and have an increased incidence after antibiotic treatment [6,8]. Principles of antimicrobial stewardship should be applied by selecting the appropriate antibiotics targeting a spectrum that effectively covers common gut bacteria and their corresponding resistance patterns, while ensuring adequate drug levels at the site of infection. It is recommended to de-escalate therapy as soon as culture results become available and stop therapy once there is adequate source control [6]. Due to challenges in achieving effective microbial control, Klebsiella pneumoniae developed resistance over time, as evidenced in the sensitivity tests of the culture samples from the necrotic collection. Microbiology results, along with antibiotic susceptibility tests and the need for multiple endoscopic procedures to control the infection focus, facilitated appropriate antibiotic de-escalation and ultimately improved the patients' outcome. 

Only 28% of patients with infected necrotizing pancreatitis are successfully treated with antibiotics alone [14]. Therefore, effective management often requires the use of endoscopic drainage to control the infectious focus. Endoscopic drainage is safer and more efficient for pancreatic fluid collections with higher clinical success, lower mortality rate, hospital stay, and re-interventions compared with percutaneous drainage [8]. Timing of intervention remains to be studied, but no significant differences were observed in clinical outcomes and patients’ mortality rate in early (<4 weeks) or standard (≥4 weeks) drainage of walled-off pancreatic fluid collections [15,16]. Percutaneous catheter drainage is a non-endoscopic option, where the most common complication is internal and external pancreatic fistulas, and the mortality rate is 17.4% [10]. Open necrosectomy is confirmed to be the last resort, useful in selected severe cases where other minimally invasive approaches failed [17].

## Conclusions

In conclusion, this case demonstrates the intricate balance needed between antimicrobial therapy and procedural interventions in managing complex pancreatic infections. A multidisciplinary team consisting of gastroenterologists, general surgeons, and intensive care physicians plays a crucial role in the selection of individualized treatment strategies in the management of severe cases of infected NP and septic shock. Given the complexity of managing infected NP, further studies are needed to establish the optimal management approach for this condition.
